# Improving quality of care for children with asthma by learning with an interactive approach: a prospective randomized controlled study in 14 Swedish primary health care centers

**DOI:** 10.1186/1710-1492-6-S4-A13

**Published:** 2010-12-10

**Authors:** Maria Ingemansson, Marina Jonsson, Gunilla Hedlin

**Affiliations:** 1 Department of Pediatric Allergy and Pediatric Outpatient Clinic, Astrid Lindgrens Hospital, 171 76 Stockholm, Sweden. Karolinska Institutet, Department of Woman's and Children's Health, 171 76 Stockholm, Sweden; 2 Department of Pediatric Outpatient Clinic, Astrid Lindgrens Hospital, 171 76 Stockholm, Sweden. Karolinska Institutet, Department of Woman's and Children's Health, 171 76 Stockholm, Sweden; 3 Department of Pediatric Allergy, Astrid Lindgrens Hospital, 171 76 Stockholm, Sweden, Karolinska Institutet, Department of Woman's and Children's Health, 171 76 Stockholm, Sweden

## 

Eight to 10% of children in Sweden have asthma. The majority of these children are followed and treated in primary health care centers (PHCs). Many studies show poor compliance to written guidelines [1,2]. The purpose of this study was to evaluate whether or not the method of interactive case-based education [3] combined with audit/feedback [4], leads to a better quality of care for children with asthma, according to quality indicators from Local Practice Guidelines (LPG) [5].

We collected data from children aged 6 months to 16 years with asthma, obstructive bronchitis or cough, attending 14 PHCs in northern Stockholm. Medical records of 20 children diagnosed with asthma were randomly selected in each PHC. Medical records of 20 children diagnosed with obstructive bronchitis or cough were also included to evaluate if these children fulfilled the diagnostic criteria of asthma. A questionnaire was addressed to general practitioners (GPs) and nurses regarding knowledge and competence in asthma management, educational needs and interest, and current equipment and routines at their PHC.

The 14 PHCs were matched by pairs. Seven PHCs received the educational intervention, while 7 PHCs had this intervention only after completion of the study. Medical records were scrutinized in order to identify the following quality indicators: diagnosis on the basis of the criteria delineated in the LPGs: performance of spirometry, assessing exposure to tobacco smoke, prescription of inhaled corticosteroids, patient education and routine follow-up. The second evaluation of the medical records and questionnaire were scheduled six months after the intervention, to allow time to change behaviour. GPs and nurses from the intervention group participated in three interactive educational meetings [3] led by an allergologist and a nurse. The first two meetings used case-based learning approaches, focusing on the specific needs of each PHC and the third meeting was based on audit/feedback [4] to discuss the results obtained and address the problems identified in the first analysis of data from the participating PHCs.

Preliminary baseline data reveals that 50% of children with asthma from school age are treated with inhaled corticosteroids, prescribed by the GPs. Few patients had a spirometry test and few had received asthma education. Exposure to tobacco smoke was rarely discussed. Most children had only one visit to the PHC. Fifty per cent of the children had a planned follow-up, usually by referral to the local paediatric outpatient clinic. Twenty per cent of the preschool children had under-diagnosed asthma.

This initial analysis shows that there are many care gaps in children’s asthma management, and that educational interventions have the potential to help address these gaps.
